# Assessing models for genetic prediction of complex traits: a comparison of visualization and quantitative methods

**DOI:** 10.1186/s12864-015-1616-z

**Published:** 2015-05-22

**Authors:** Sarah A. Gagliano, Andrew D. Paterson, Michael E. Weale, Jo Knight

**Affiliations:** Campbell Family Mental Health Research Institute, Centre for Addiction and Mental Health, Toronto, Ontario Canada; Institute of Medical Science, University of Toronto, Toronto, Ontario Canada; Department of Psychiatry, University of Toronto, Toronto, Ontario Canada; Program in Genetics and Genome Biology, The Hospital for Sick Children, Toronto, Ontario Canada; Biostatistics Division, Dalla Lana School of Public Health, University of Toronto, Toronto, Ontario Canada; Epidemiology Division, Dalla Lana School of Public Health, University of Toronto, Toronto, Ontario Canada; Department of Medical & Molecular Genetics, King’s College London, Guy’s Hospital, London, UK

**Keywords:** Predictive accuracy, Genetic prediction, Receiver operating characteristic curve

## Abstract

**Background:**

*In silico* models have recently been created in order to predict which genetic variants are more likely to contribute to the risk of a complex trait given their functional characteristics. However, there has been no comprehensive review as to which type of predictive accuracy measures and data visualization techniques are most useful for assessing these models.

**Methods:**

We assessed the performance of the models for predicting risk using various methodologies, some of which include: receiver operating characteristic (ROC) curves, histograms of classification probability, and the novel use of the quantile-quantile plot. These measures have variable interpretability depending on factors such as whether the dataset is balanced in terms of numbers of genetic variants classified as risk variants versus those that are not.

**Results:**

We conclude that the area under the curve (AUC) is a suitable starting place, and for models with similar AUCs, violin plots are particularly useful for examining the distribution of the risk scores.

**Electronic supplementary material:**

The online version of this article (doi:10.1186/s12864-015-1616-z) contains supplementary material, which is available to authorized users.

## Background

The risk of developing a complex trait is influenced by many genetic variants, possibly hundreds, in combination with environmental factors. Genome-wide association studies (GWAS) have had success in identifying some of the genetic risk factors involved in complex traits, but more remain to be discovered. Recently, there have been several *in silico* attempts at utilizing epigenetic and genomic data to prioritize genetic risk variants. These methods simultaneously incorporate multiple lines of genomic and epigenomic data to identify potential risk variants from all variants [1-6]. These data tend to have the characteristic of consisting of imbalanced classes: a very high proportion of non-risk variants (“non-hits”) and a small proportion of risk variants (“hits”). This class imbalance, and other factors unique to genetic data (for instance linkage disequilibrium, allele frequency, etc.), warrant exercising caution when interpreting the results of predictive accuracy measures that are applied to such models.

A variety of predictive accuracy measures and data visualization techniques have been used (Table 1) to assess these models for prioritizing genetic variants. An example is the area under the curve (AUC) from the receiver operating characteristic (ROC) curve, which is generally accepted as a measure of how closely the prediction values reflect the true class. Such methods have previously been employed to predict diagnosis of an individual (risk of developing Type II Diabetes [7-9], for example), but have only recently been applied to predict whether genetic variants are likely to be risk variants.

**Table 1:** Predictive accuracy measures in the literature for models for prediction of variants associated with complex traits

			Predictive accuracy measures employed					
	Algorithm	Classifier	Area under ROC curve	Positive predictive value	Box plot	Histo-Gram	Violin plot	Mann–Whitney U/Wilcoxon Rank Sum test
Gagliano et al. 2014	Modified Elastic net	GWAS hits vs. non-hits	x	x		x		
Iversen et al. 2014	Penalized logistic regression	GWAS hits vs. non-hits	x*					
Kircher et al. 2014	Support Vector Machines	High-frequency human-derived alleles vs. simulated variants	x				x	x
Ritchie et al. 2014	Modified Random Forest	HGMD hits vs. non-hits	x		x			x

*reports “Concordance index”, which is equivalent to the area under the ROC curve

We will utilize test set data from a regularized logistic model that predicts genetic risk variants on the basis of a large multivariate functional dataset [1]. We investigate the utility of several approaches for assessing predictive accuracy and data visualization. Based on observations from this work we conclude with suggested guidelines to aid researchers when assessing models for genetic variant prediction.

Three broad categories of predictive accuracy measures will be discussed: (1) concepts in describing predictive accuracy, including ROC, AUC and the confusion matrix (2) visualization of the distribution of prediction values, and (3) statistical tests. All the methods described below were conducted in R, version 3.0.2 [10-13]. See Table 2. Sample R code is available in Additional file 1. Code and data to reproduce the results in this paper are provided in Additional file 2. Further details are embedded in the results.

**Table 2:** Predictive accuracy measures and the corresponding R package in which they can be computed

Predictive accuracy measure	R package	Version
(1) The confusion matrix		
Receiver Operating Characteristic Curve and area under the curve	prediction and performance in ROCR [11] performance (prediction.object, “auc”)	1.0-7
Positive predictive value and negative predictive value	prediction and performance in ROCR performance (prediction.object, “ppv”) performance (prediction.object, “npv”)	1.0-7
(2) Visualization of the distribution of prediction values		
Histograms of the prediction values separated by class	multhist in plotrix [12]	3.5-11
Box plots	boxplot in graphics	Base package
Violin plots	vioplot in vioplot	
Quantile-quantile plots	qqplot in stats	Base package
(3) Statistical tests		
Hypergeometric test	phyper in stats	Base package
Mann–Whitney *U* test	wilcox.test in stats	Base package
Asymptotic Generalized Cochran-Mantel-Haenszel Test	cmh_test in coin [13]	1.0-24

## Methods

### Dataset and models

The example dataset and model have been described in detail previously [1] and are only described briefly here. Genetic variants from common genotyping arrays were annotated for 14 functional characteristics (twelve of which are binary and two are quantitative), many of which are from the Encyclopedia of DNA Elements (ENCODE) Project, with data from various cell types merged (un-weighted) into a single variable for each characteristic. All functional characteristics could be presented in a binary presence/absence format with the exception of two types conservation scores, which remained on a quantitative scale. A regularized logistic model, capable of handling correlated predictor variables, was used. A random 60 % of the genetic variants were assigned to the training set to determine the parameters of the model, and the remaining variants were reserved for the independent test set to evaluate the accuracy of the model. All models produced a prediction value ranging from 0 to 1 for each genetic variant, with values close to 1 implying high probability of the variant contributing to risk. Due to the unbalanced nature of the data a weighting procedure that equalizes the importance of hits and non-hits in the training set was employed. Hits were weighted by (N_hits_ + N_non-hits_)/2N_hits_ and all non-hits by (N_hits_ + N_non-hits_)/2N_non-hits_, where N_hits_ and N_non-hits_ denote the number of hits and non-hits, respectively, in the training set [1]. Without this weighting scheme, all variants are assigned low prediction values although the model still retains comparable overall accuracy. Overall accuracy may not be representative of accuracy within classification groups, which is the main problem with unbalanced data. As well as using the weighting scheme to ameliorate this issue in our example data we discuss other matters to be considered in relation to the accuracy and data visualization methods described.

For model 1, variants were classified as being hits if present in the genome-wide association study (GWAS) Catalogue published by the National Human Genome Research Institute [14] downloaded on August 6, 2013. The GWAS Catalogue reports variants found to be associated with disease or quantitative trait in a GWAS study with a p-value <1x10^−6^. Variants not present in the Catalogue but present on common genotyping arrays were assumed to be non-hits. Three alternate classifiers were used to designate hits: (a) p-value < 5x10^−8^ (model 2), and (b) p-value < 5x10^−8^ for only a subset of phenotype specific hits namely an autoimmune (model 3) and a brain-related analysis (model 4).

In our previous work, six models were created using the alterations to the classifier described above. The four assessed here are the two models with the highest AUC (models 2 and 3) and two models with the lowest AUC (models 1 and 4). (See Table 3 for descriptive statistics for the test sets of the various models).

**Table 3:** Descriptive statistics for the various genetic prediction models from Gagliano et al. (2014) to be used as examples here

Phenotype-specific analyses		N	Minimum	25 % percentile	Median	Mean	75 % percentile	Maximum	Standard deviation	N outliers*
Brain-related	Hits	144	0.40	0.42	0.51	0.51	0.57	0.77	0.09	3
	Non-hits	32723	0.40	0.40	0.46	0.48	0.53	0.79	0.07	61
Autoimmune	Hits	234	0.29	0.45	0.55	0.55	0.66	0.86	0.14	0
	Non-hits	33266	0.29	0.30	0.44	0.45	0.55	0.93	0.13	0
All phenotype analyses										
p < 5E-8	Hits	1292	0.32	0.44	0.54	0.54	0.62	0.92	0.13	4
	Non-hits	30135	0.32	0.35	0.44	0.46	0.55	0.91	0.12	7
all GWAS Catalogue	Hits	3405	0.44	0.45	0.50	0.51	0.54	0.81	0.06	144
	Non-hits	30039	0.44	0.44	0.48	0.49	0.52	0.80	0.05	336

*Outliers are defined as data points outside 1.5x interquartile range (interquartile range = 75 % percentile - 25 % percentile)

Ethical approval was not required for this study.

## Results

### Concepts in describing predictive accuracy

#### The confusion matrix

**Fig. 1:**
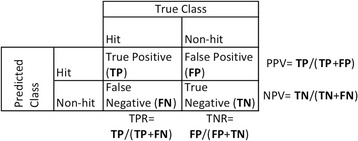
A Confusion matrix and its relation to predictive accuracy terms. TPR = True Positive Rate, TNR = True Negative Rate, PPV = Positive Predictive Value, NPV = Negative Predictive Value

Predictive accuracy is derived from a confusion matrix (Fig. 1). The cells in the diagonal of the matrix are the correctly identified genetic variants. (See Chapter 4 in “*An Introduction to Statistical Learning with Applications in R*” [15] and Chapter 11 in “*Statistical Learning for Biomedical Data*” [16] for more details.) The effects of unbalanced data in un-weighted models can be detected in such a matrix. There would be a much larger proportion of negatives compared to positives. The effects on false positive rate (FPR), true negative rate (TNR), positive predictive value (PPV), and negative predictive value (NPV) are described in further detail below. The confusion matrix itself is not often studied as it represents data at only one threshold. However both the ROC curve and PPV and NPV are used to consider model accuracy.

#### Receiver operating characteristic curves and area under the curve

The use of ROC curves is a common way for assessing binary outcome models [17]. ROC curves offer a global summary of machine performance at all possible cut-offs of prediction values for defining the two classes. In this way, the ROC is a summary of the model’s overall performance. ROC curves reflect the columns of the confusion matrix by presenting FPR (equivalent to 1-TNR)) by true positive rate (TPR), with the advantage of depicting these values at every threshold for defining a hit. An AUC = 0.5 means that the predictive accuracy of the model is not better than chance, whereas an AUC = 1 implies perfect predictive accuracy. (See Chapter 4 in “*Road to Statistical Bioinformatics*” [18] and Chapter 11 in “*Statistical Learning for Biomedical Data*” [16] for more details).

There typically is not just one confusion matrix (see previous section), but rather there is an infinite number: one for each point along the x-axis of the ROC. Thus in the context of a model that outputs prediction values measured on a continuous scale rather than binary categories (e.g. a logistic regression model among others) one needs to decide at what probability level one “declares” a hit to be a hit. One could use the arbitrary value of greater than 0.5 as the cut-off to declare hits from non-hits, but there are other probability thresholds one could use, which can be summed up in a ROC curve. That is the conceptual difference between the AUC (average over all possible thresholds) and the confusion matrix itself (considers the ROC “frozen” at one particular probability threshold).

It should be noted that unless a weighting scheme such as the one we employed in our modeling or an equal subset of both classes is chosen, ROC curves can present an overly optimistic view of performance for unbalanced data [17]. If the model simply assigns all variants to the non-hit class then it will appear to do well, for instance with an AUC much larger than 0.5. In this way, the larger class (non-hits) can overwhelm the smaller class (hits). The TPR thus tends to be low throughout the thresholds.

**Fig. 2:**
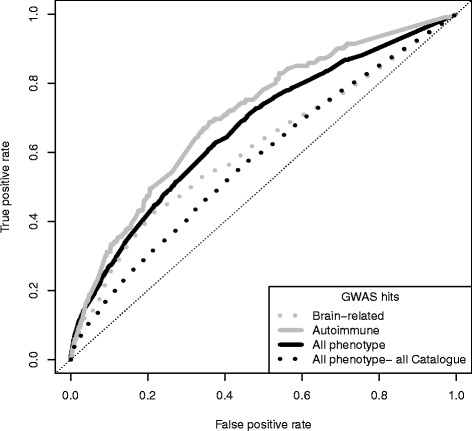
ROC curves for the four models

In the example data, the AUC of two of the models (autoimmune and all phenotype for the high confidence hits) were very similar and reasonably good (between 0.67 and 0.71) (see Fig. 2). The AUC for the other two models (the all phenotype using all Catalogue hits and the brain-related models) were also similar to each other, but poor (less than 0.61). Thus, the AUC seems to categorize models as either good or poor, but is not particularly useful for finer discrimination between models. (See Chapter 11 in “*Statistical Learning for Biomedical Data*” [16] for details on the limitations of ROC curves.) Below we demonstrate that additional investigation provides further insight into the results.

#### Positive and negative predictive values

The rows of the confusion matrix are represented by PPV and NPV. PPV is the probability of variants that are true hits being correctly classified as hits, and NPV is the probability of variants that are true non-hits being correctly classified as non-hits at any one given threshold. (See Chapter 4 in “*Road to Statistical Bioinformatics*” [18] for details.) PPV and NPV are also affected by the class imbalance inherent in real genetic association data. The effect of imbalanced data on PPV and NPV has been previously described [19]. In scenarios where the negative class is larger than the positive class, NPV is inflated and PPV is lower compared to the corresponding model where the class sizes are equal and the negative and predictive classes have the same rate of correct predictions [19]. These values are best when there are equal amounts of data in each category [19]. The issue is that cell sizes of the confusion matrix can become too small for the smaller class (hits). One needs to ensure that there is a large enough quantity of hits and/or non-hits per cell in the confusion matrix to draw conclusions. Otherwise, results will be driven by a very small unrepresentative subset of the data. For the models considered here, only the two all phenotype analyses had an adequate amount of samples in each cell, and thus PPV and NPV were only calculated for those models. The NPV tended to be high (>0.899) at all the various prediction value thresholds chosen to define the two classes. See Table 4. However, it is the accuracy of predicting the hits, not the non-hits, which is of interest in this work. Hence, the PPV provides more interesting results. Overall, the all phenotype analysis using all hits in the GWAS Catalogue produced the highest PPVs as the threshold for declaring a positive hit increased. The highest PPV (30.4 %) was achieved for this model at the threshold defining hits as those variants with prediction values greater than 0.7. PPV results conflict between the AUC results. For the two all phenotype models, the one with the higher AUC (the model for the GWAS hits in the Catalogue with the stringent p-value cut-off) had overall lower PPV compared to the model using all GWAS hits in the Catalogue. NPV results for the two models were similar, but the model based on all GWAS hits in the Catalogue had slightly lower NPV compared to the stringent p-value model.

**Table 4:** Positive predictive and negative predictive values at various prediction value cut-offs for the two all phenotype analyses

	Positive predictive values		Negative predictive values	
Prediction value cut-off	p < 5E-08 hits	all GWAS hits in catalogue	p < 5E-08 hits	all GWAS hits in catalogue
0.5	0.069	0.128	0.968	0.915
0.6	0.094	0.226	0.956	0.903
0.7	0.198	0.304	0.948	0.899

### Visualization of the distribution of prediction values

#### Histograms

**Fig. 3:**
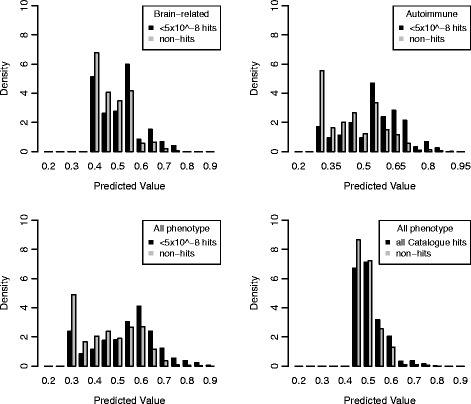
Histogram of predictive values for the all phenotype models with a bin size of 0.05. Compare to Fig. 4 with a bin size of 0.1. For the probability densities, the sum of the area under the black bars adds up to one. The same is true for the grey bars. The ideal plot would have two non-overlapping distributions with the distribution of the grey bars closest to 0 and the distribution of the black bars close to 1

**Fig. 4:**
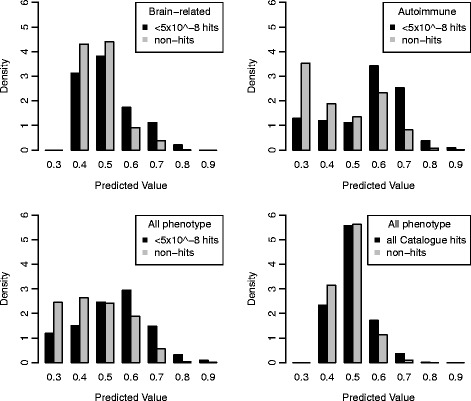
Histogram of predictive values for the all phenotype models with a bin size of 0.1. Compare to Fig. 3 with a bin size of 0.05. For the probability densities, the sum of the area under the black bars adds up to one. The same is true for the grey bars. The ideal plot would have two non-overlapping distributions with the distribution of the grey bars closest to 0 and the distribution of the black bars close to 1. The bin size is 0.1

Next, class separation was investigated through histograms of the prediction values outputted from the models, which display differences in the density distribution between the two classes. Known hits were plotted in black and non-hits in grey on the same plot, with the y-axis being probability densities, rather than numerical quantity, which masks the data imbalance and thus allows for comparison between the two classes. The all phenotype model with high confidence hits (Fig. 3) and the autoimmune model showed the most evidence of having two separate distributions. Although the distributions of the prediction values for the hits and the non-hits overlap, the distribution of the non-hits has the majority of its values closer to the 0 end of the prediction value range. Confirming the AUC results, the brain-related model and all phenotype model using all Catalogue hits (Fig. 3) do poorly with regard to class separation. As always, caution is warranted since the visualization of the distributions differ depending on the bin size chosen (compare Fig. 3 to Fig. 4). For the histograms with a larger bin size differences in distributions between hits and non-hits at a finer scale is less apparent, and the distributions look more similar compared to if a smaller bin size is used.

#### Box and whisker plots

**Fig. 5:**
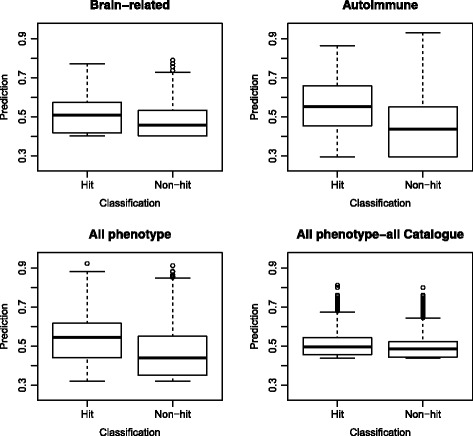
Box and whisker plots for the four models. The line in the box is the median, and the box outlines the 25 % and 75 % percentiles. Outliers are shown as individual data points if the value is 1.5 times the interquartile range (IQR). The lower and upper whiskers on the plot represent the 25 % percentile minus 1.5*IQR and the 75 % percentile plus 1.5*IQR, respectively. If the data does not extend as far as those calculated ranges, then the whisker is plotted at the value of the minimum or maximum data point

Box plots were constructed to visually compare the distributions of the hits versus the non-hits in an alternate way (Fig. 5). These plots visually depict much of the descriptive data present in Table 3, notably differences in the median between the two classes. Again the data imbalance is masked as the summaries presented in the plot are from within each class. As visualized in the histograms, the box plots also showed that for all of the models the distributions of the prediction values for the hits and non-hits overlapped, but to different degrees. The plots for the brain-related model and the all phenotype model for all variants in the GWAS Catalogue had many outliers for both classes, signifying that for both hits and non-hits had predictions that were a large distance from the predictions of other variants in the respective class. Additionally, the mean prediction scores for the hits and the non-hits appear very close for the all phenotype model for all variants in the GWAS Catalogue.

#### Violin plots

**Fig. 6:**
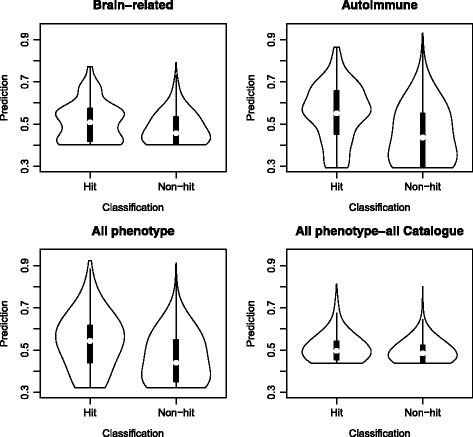
Violin plots of the four models

Violin plots visually combine the density differences depicted in the histograms and the median differences depicted in the box plots into one plot. These plots summarize the results of the histograms and box plots. Furthermore, they are comparable to a histogram with infinitely small bin sizes. See Fig. 6.

#### Quantile-quantile plots

**Fig. 7:**
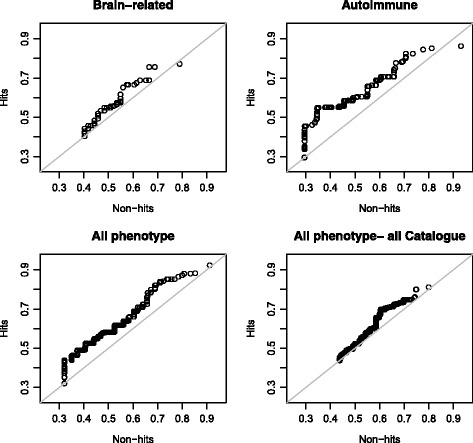
Quantile-quantile plots for the four models

A final visualization method, the quantile-quantile plot was explored. See Fig. 7. The quantile-quantile plot is often used in the context of GWAS, but it also has the potential to be useful as a predictive accuracy measures. Instead of expected and observed p-values on the axes as what is done in GWAS, we plotted prediction values for non-hits on the x-axis and prediction values for the hits on the y-axis. Plotted in this way, the plot compares the quantiles of the hits to the non-hits. When the data points on the plot deviate above the diagonal, the hits have higher prediction values compared to non-hits in that quantile. Due to a limited number of hits, the quantile-quantile plots for the phenotype-specific analyses produced a staircase pattern. This pattern suggests two characteristics: those models are assigning the same prediction value to several variants, and also there are not enough hits to create a smooth curve. The former could be due to there being different variants that have been assigned identical or similar functional characteristics. The models are binning variants together and are not able to differentiate them on a finer scale. The small sample size for the phenotype specific analyses, makes it difficult to draw conclusions from those quantile-quantile plots. For the two all phenotype analyses, the quantile-quantile plots supported the findings from the other visualization methods that the high confidence all phenotype analysis separated hits from non-hits better than the analysis based on hits from the GWAS Catalogue. For the all phenotype model based on the high confidence hits, the distribution consistently deviated from the diagonal. The distribution demonstrates that the hits had higher prediction values than non-hits in the same quantiles. The all phenotype analysis based on all hits in the GWAS Catalogue produced a quantile-quantile plot that closely followed the line for prediction values less than 0.6. This group of prediction values contained most of the data since from the histograms it was determined that the distribution of the prediction values is skewed so that most of the data fall in the lower percentiles. The distribution deviated from the diagonal roughly in the prediction value range of 0.6 and 0.7.

### Statistical tests

#### Hypergeometric test

The hypergeometric test was also used to identify significant enrichment of hits compared to non-hits in particular prediction value bins by splitting the data into bin sizes of 0.05 ranging from less than 0.35 up to 0.95. For each model, there were effectively 13 tests performed, one test per prediction value bin. Based on this resulting contingency table, significant enrichment of hits was seen for all of the models in at least one bin greater than 0.55 (with significant p-values ranging from 0.01 to 5.58×10^−29^), while no enrichment (all p-values greater than 0.2) was seen in bins less than 0.55.

#### Cochran-Mantel-Haenszel test

Another test was investigated, the asymptotic generalized Cochran-Mantel-Haenszel test, which tests the independence of two possibly ordered factors (prediction values of hits vs. non-hits). As with the hypergeometric, a contingency table for hits and non-hits stratified by prediction value was created. Hits and non-hits were stratified independently by prediction values by splitting the data into bin sizes of 0.05 ranging from less than 0.35 up to 0.95. Rather than a single test per prediction value bin as in the hypergeometric, the generalized Cochran-Mantel-Haenszel test is a single omnibus test per model. It looks for a trend across the span of prediction values. Similar to the other statistical tests explored in this section, significant p-values were produced for all models (p < 5.3x10^−9^).

#### Mann–Whitney *U* test

A two-sided Mann–Whitney *U* test can be used to determine whether or not the distributions of the prediction values for the hits differs significantly from that of the non-hits. The Mann–Whitney U tests whether the ranks of the variants in the hit and non-hit sets differ. Significant p-values were obtained for all analyses, including those with poor AUCs and poor class separation; most notably the all phenotype analysis not refined to the high confidence hits had a Mann–Whitney p-value of 7.17x10^−50^. It was hypothesized that this significant p-value was due to the class imbalance and/or outliers. To explore these hypotheses, only a random subset of non-hits equal in size to the number of hits were selected for the Mann–Whitney *U* test, and in other test only outliers were removed. In both situations, the p-values tended to remain highly significant (Table 5).

**Table 5:** Mann–Whitney U p-values for the four models

Mann Whitney U p value			
	Unaltered	n(hits) = n(nonhits)	No outliers (1.5x outside 25 % or 75 % percentiles)
Phenotype-specific analyses			
Brain-related	3.49E-06	0.007447	1.76E-05
Autoimmune	8.63E-28	5.26E-15	8.63E-28
All phenotype analyses			
p < 5E-8	2.08E-93	3.01E-52	3.53E-92
All Catalogue	7.17E-50	7.26E-27	1.37E-34

**Fig. 8:**
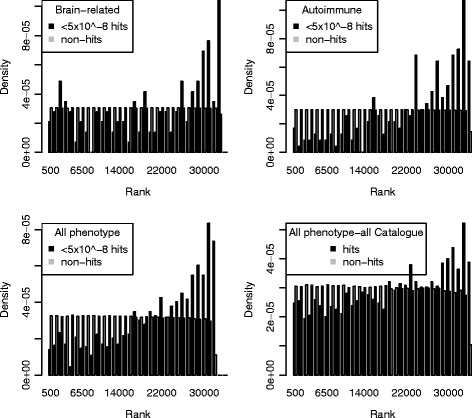
Ranked Mann–Whitney U p-values plotted separately for the hits and non-hits. The non-hits follow a uniform distribution, whereas the hits do not. The same pattern was observed for all four models

The significant Mann–Whitney U p-values do not necessarily suggest that the hits and non-hits are well separated by their prediction values. Instead, the p-values are highlighting differences in ranks between the hits and the non-hits, which may or may not imply class separation. We plotted the hits and non-hits according to their ranks. In all of the plots, the non-hits follow a uniform distribution, whereas the hits follow a different distribution, roughly negatively skewed (Fig. 8). Thus, as with enrichment according to the hypergeometric, and the Cochran-Mantel-Haenszel test for independence, differences in rank according to the Mann–Whitney U are not particularly informative with regard to class separation between the hits and non-hits according to their prediction values.

The statistical tests mentioned above do not explicitly measure class separation between hits and non-hits based on their prediction values, which is a key outcome for investigating the predictive accuracy of models for variant prioritization. The hypergeometric assesses enrichment of hits, the Mann–Whitney U tests for differences in ranks between the hits and non-hits, and the generalized Cochran-Mantel-Haenszel test evaluates independence of the hits and non-hits. Thus, significant p-values from these statistical tests cannot alone be taken as proof of class separation or model performance.

## Discussion

In this review we summarized various predictive accuracy measures related to the confusion matrix, visualization methods, and some statistical tests. These methods were described in the context of genetic models for prediction of risk variants in complex traits in which a class imbalance between the hits and non-hits is often inherent.

The choice of predictive accuracy measures was partially motivated by the measures found in the publications described in the background as well as other measures. Note that two of the mentioned papers, [3,5], both focused on investigating enrichment or depletion of disease- or trait-associated variants with particular functional and genomic features. Since the predictive accuracy measures in those papers did not relate to an output of a prediction value for each variant, those methods were not discussed further.

In summary, the investigation above emphasizes the importance of visualizing the underlying distributions of the classes. The ROC curve is a good starting place, but visualization measures, especially violin plots, are valuable for differentiating models with similar AUCs. A downside of histograms is that depending on the bin size, the interpretation of the results may vary. With regard to box plots, these plots do not offer any information about density. On the other hand, violin plots are able to show density without the need of binning and at the same time depict the summary statistics that would be seen from a box plot (for instance, [20]). Caution is needed when making conclusions about model performance based on p-values, such as from the Mann–Whitney *U* test. Significant p-values cannot necessarily be attributed to a good separation between hits and non-hits. Visualizing the class distribution seems to be the most informative for determining the predictive accuracy in these scenarios.

## Conclusions

All of the papers mentioned in the introduction apply their model(s) to real data to assess the accuracy of identifying disease-relevant genetic variants. Predictive accuracy measures and visualization of the prediction values can only show model performance in theory. When evaluating model performance it is also vital to assess the model in real applications.

## Additional files

Additional file 1:
**R: Sample R code to perform the tests mentioned in this paper.** MyData.txt: Sample output data from a model on which to run the code. Additional file 2:
**Code-for-paper.** R: R code to reproduce the results in this paper. Autoimmune-testset.csv, Brain-testset.csv, Nonpheno-5e-8-testset.csv, Nonpheno-allCat-testset.csv: data files required for Code-for-paper. R; they contain five columns: the identifier for the genetic variant, base position, chromosome number, the classifier (hit = 1, non-hit = 0), and the prediction value.

## Abbreviations

AUC: Area under the curve
FNR: False negative rate
FPR: False positive rate
GWAS: Genome-wide association study
HGMD: Human gene mutation database
NPV: Negative predictive value
PPV: Positive predictive value
ROC: Receiver operating characteristic curve
TNR: True negative rate
TPR: True positive rate
